# The impact of COVID-19 mRNA vaccination on somatic and psychiatric symptoms in patients with treatment-resistant schizophrenia: a cohort study

**DOI:** 10.1192/j.eurpsy.2024.1071

**Published:** 2024-08-27

**Authors:** A. Touiti, I. Yaich, C. Ben Said, N. Bram

**Affiliations:** ^1^Forensic Psychiatry Departement, Razi Hospital, La Manouba; ^2^Faculty of Medecine of Tunis, Tunis El Manar University, Tunis, Tunisia

## Abstract

**Introduction:**

Patients with mental illness, particularly those with treatment-resistant schizophrenia, are at increased risk of severe COVID-19. The protective effect of vaccination against severe disease has been demonstrated, and vaccination of vulnerable individuals was a priority during the vaccination campaign. However, the effect of vaccination on the psychiatric symptoms of the disease is not well understood.

**Objectives:**

To investigate the impact of COVID-19 vaccination on psychiatric symptoms and somatic symptoms in patients hospitalized for treatment-resistant schizophrenia.

**Methods:**

Thirty patients hospitalized for treatment-resistant schizophrenia with a history of medico-legal acts were admitted to the forensic psychiatry department at Razi Hospital in Manouba, Tunisia. The consent of patients and/or their relatives was obtained before vaccination, and potential side effects were explained to patients and their families. A neuropsychiatric assessment and clinical examination of patients were performed by their referring psychiatrist before vaccination and one month after.

**Results:**

The patients were all male, with a mean age of 42.3 years.No patient had an allergic reaction to the vaccine. No patient was infected with the virus one month after vaccination. On the clinical level, 30% of patients had general symptoms such as fatigue and myalgia, which improved spontaneously within a few days. On the psychiatric level, exacerbation of positive symptoms such as hallucinations and delusions was found in 26% of patients. No increase in the frequency of agitation episodes or risk of hetero-aggressive behavior was reported. Sleep disturbances such as difficulty falling asleep and fragmented sleep were reported. The most common functional complaints reported by patients were palpitations, which were a source of somatic concern.

**Conclusions:**

Several side effects of the vaccine have been documented and are taken into account in the daily practice of practitioners, but psychiatric effects are poorly reported and are sometimes attributed to the underlying disease. A complete examination, objective assessment, and regular follow-up are necessary to identify symptoms early and prevent relapses.Because of the small size of the sample;results could not be generelized.Further studies on a larger scale should be conducted.

**Disclosure of Interest:**

None Declared

